# The Status Lymphaticus and the Ductless Glands1Read at the annual meeting of the American Surgical Association, San Francisco, Cal., July 5, 1905.

**Published:** 1905-08

**Authors:** Roswell Park

**Affiliations:** Buffalo, N. Y., Professor of Surgery at the University. 510 Delaware Avenue


### The Status Lymphaticus and the Ductless Glands.[1]

By ROSWELL PARK, M. D., LL.D., Buffalo, N. Y.,
Professor of Surgery at the University.

(*Author's Abstract.*)

THE writer defined the term status lymphaticus and stated that the reason for bringing the subject before a body of surgeons was because the condition itself had so much to do with the question of toleration of anesthetics, and the matter of repair of wounds, that it really was one of exceeding importance. Not a few cases of sudden death during and after operation were to be explained by the abnormalities constituting this condition. These have usually been attributed to the anesthetic or the anesthetist, and it may be that had the latter appreciated all the dangers offered by the condition he would have guarded his anesthetic better or perhaps abstained from giving it. Nevertheless, the anesthetiser is not always to blame, because he is frequently called in without previous knowledge of the case, it being really the function of the medical attendant to carefully study it and determine its peculiarities.

The relation which the lymphatic system and the lymph-making organs bear to each other and to the ductless glands is as positive as it is obscure. Lymphoid tissue throughout the body seems to increase as the thymus normally disappears. In infantile marasmus the thymus has usually completely and prematurely disappeared, its condition being a fair index as to general nutrition. But it is hypertrophy rather than atrophy of the thymus which most interests the surgeon, since those individuals who die suddenly and unexpectedly usually have an enlarged thymus. There certainly exist strange relationships between the pituitary body, the thymus, the thyroid, the coccygeal body (to which too little attention has been paid), the testes, the ovaries, and perhaps the bone marrow. In this list the quite recent researches relative to the parathyroids should also be included. Indeed, a clearer recognition of the function of the latter seems to have changed the whole aspect in which we should regard Graves's disease, it being made quite probable that this condition is due rather to atrophy of the parathyroids than to excess of the thyroid or its secretion. In the ordinary forms of cystic goiter, where thyroid secretion is not augmented, the symptoms of Graves's disease are lacking. It is usually after removal of the thyroid proper that the opposite symptoms of Graves's disease

1. Read at the annual meeting of the American Surgical Association, San Francisco, Cal., July 5, 1905.

appear, that is, myxedema and cachexia, while when the parathyroids are taken away the peculiar symptoms of Graves's appear.

The life history of the thymus is closely related to that of the testes; atrophy of the former seems to depend upon complete maturation of the latter. These statements can be corroborated by a study of cryptorchids and castrated animals.

The status lymphaticus is also described under the terms lymphatism, lymphatic constitution and status thymicus, the condition having apparently more to do with the thymus than with any other isolated organ. Thymus extract produces in animals a fall of blood pressure with acceleration of the heart, and, in fatal doses, collapse. Thymic enlargement may cause death by pressure on important structures, even distinct from the trachea. Thymic asthma, a condition known for some time, is one in which death sometimes occurs most unexpectedly and quickly. In these cases not only is the thymus enlarged, but there is found a general hyperplasia of the lymphatic tissue, with enlargement of the lymph nodes and of the spleen.

The relations between status lymphaticus and rickets are frequent and pronounced. Nearly all cases of the former display the ordinary clinical evidences of the latter. The participation of the lymphatic system is most casually apparent in the involvement of the faucial, laryngeal and lingual tonsils; these are the so-called adenoids of the throat specialists. It is notable that it is in these cases that death has most often occurred during or after anesthesia.

Thymic asthma is practically the same thing as laryngismus stridulus. The occurrence of this in children ought always to serve as a warning, as well as an indication for careful study and treatment. Should, then, chloroform be given to such a case inadvertently or unnecessarily, the agent would not be nearly as responsible for the death as the practitioner who employs it.

The condition is to be recognised by the ordinary evidences of rickets which are usually present, by generalised enlargement of the lymph nodes, the presence of adenoids in the nasopharynx, a history of laryngismus stridulus or special liability to spasm of the glottis, perceptible enlargement of the thymus or by notable enlargement of the thyroid or spleen. In the young these conditions simulate cretinism.

Such a clinical picture as will afford the above features should serve as a warning and make one abstain from the use of anesthetics unless absolutely necessary, while it should also prompt carefully directed treatment. This treatment should consist in careful attention to elimination, as well as to nutrition, while thymus or pituitary extract should be administered internally, as well as the glycerophosphates or some similar upbuilding tonic. If now it should be necessary to clear out the nasopharynx, in such a case, the parts should be first desensitised by cocaine, to which a little adrenalin may be added, even previous to commencing anesthesia. If the patient be known to be subject to glottic spasm one should be prepared to do a tracheotomy on an instant's notice, and especially so if the thymus be perceptibly enlarged; one might even have to use a long trachea tube. Operations of convenience, then, should be postponed until the patients can be carefully built up; operations of necessity may be practised with the above precautions, while in cases of impending or actual emergency one may depend upon artificial respiration—after tracheotomy, and the use of adrenalin.

510 Delaware Avenue.

